# Combined diet and physical activity is better than diet or physical activity alone at improving health outcomes for patients in New Zealand’s primary care intervention

**DOI:** 10.1186/s12889-018-5152-z

**Published:** 2018-02-08

**Authors:** Catherine Anne Elliot, Michael John Hamlin

**Affiliations:** Department of Tourism, Sport and Society, Lincoln University, PO Box 85084, Lincoln, Christchurch, Canterbury 7647 New Zealand

**Keywords:** Primary care intervention, Physical activity, Exercise prescription, Disease prevention, Diet, Metabolic health, Physiologic, Psychologic, Behavior change, Nutrition

## Abstract

**Background:**

A dearth of knowledge exists regarding how multiple health behavior changes made within an exercise prescription programme can improve health parameters. This study aimed to analyse the impact of changing diet and increasing exercise on health improvements among exercise prescription patients.

**Methods:**

In 2016, a representative sample of all enroled New Zealand exercise prescription programme (Green Prescription) patients were surveyed (*N* = 1488, 29% male, 46% ≥ 60 yr). Seven subsamples were created according to their associated health problems; metabolic (*n* = 1192), physiological (*n* = 627), psychological (*n* = 447), sleep problems (*n* = 253), breathing difficulties (*n* = 243), fall prevention (*n* = 104), and smoking (*n* = 67). After controlling for sex and age, multinomial regression analyses were executed.

**Results:**

Overall, weight problems were most prevalent (*n* = 886, 60%), followed by high blood pressure/risk of stroke (*n* = 424, 29%), arthritis (*n* = 397, 27%), and back pain/problems (*n* = 382, 26%). Among patients who reported metabolic health problems, those who changed their diet were 7.2, 2.4 and 3.5 times more likely to lose weight, lower their blood pressure, and lower their cholesterol, respectively compared to the control group. Moreover, those who increased their physical activity levels were 5.2 times more likely to lose weight in comparison to controls. Patients who both increased physical activity and improved diet revealed higher odds of experiencing health improvements than those who only made one change. Most notably, the odds of losing weight were much higher for patients changing both behaviours (17.5) versus changing only physical activity (5.2) or only diet (7.2).

**Conclusions:**

Although it is not currently a programme objective, policy-makers could include nutrition education within the Green Prescription initiative, particularly for the 55% of patients who changed their diet while in the programme. Physical activity prescription with a complimentary nutrition education component could benefit the largest group of patients who report metabolic health problems.

## Background

A lack of physical activity, tobacco smoking and an unhealthy diet contribute to almost 80% of the world’s risk of cardiovascular disease and type 2 diabetes [1]. Positioned as the leading cause of premature death globally [2], cardiovascular disease is an epidemic driven by type 2 diabetes and the metabolic syndrome [3]. Empirical evidence suggests that the co-occurrence of behavioral risk factors yield greater risks for chronic diseases than the sum of their individual independent effects [4, 5]. For instance, individuals who are diagnosed with metabolic syndrome show a 50-60% higher risk of having a cardiovascular disease than those without metabolic syndrome [6]. With an estimated 20-25% of the world’s adult population presenting metabolic syndrome [3], multiple disease risk factors are increasingly common in adults [7].

Major risk factors of cardiovascular disease and metabolic syndrome are physical inactivity and poor diet [8] with physical inactivity positioned as the primary cause of most chronic diseases [9]. Although compelling evidence exists for the efficacy of improving physical activity and diet [10] in treating individuals with multiple risk factors [11], usual care relies on pharmacotherapies which merely address disease symptoms [12].

Cardiovascular disease is the number one single cause of death in New Zealand, accounting for 33% per annum [13]. In 1998, New Zealand actively addressed this concern by initiating a primary-care intervention strategy called Green Prescription, whereby general practitioners and practice nurses refer or prescribe eligible patients to trained personnel [14]. Nearly 40,000 Green Prescription referrals were written by clinicians in New Zealand from 2013 to 2014 [15]. Green Prescription patients might receive an exercise prescription for any combination of cardiorespiratory, metabolic, physiological or psychological reasons. Once enroled, patients meet with physical activity specialists who customise a physical activity routine which is catered to the patients’ needs and lifestyles while addressing barriers such as asthma, injury, back pain, etc.

The Green Prescription Programme is akin to a globally adopted health initiative called Exercise is Medicine. Since both programmes focus on increasing physical activity a as means of chronic disease prevention, there is little scope to focus on the nutritional component of the energy balance equation. Nevertheless, 68% of survey respondents reported they have received information on healthy eating through Green Prescription. Additionally, 55% of patients in the subsamples analysed in this study reported changing diet as well as physical activity. From a physiological perspective, the energy balance behaviors of increasing physical activity and changing diet are major preventive therapies, particularly for weight loss, [10, 16] but also for metabolic syndrome [11] and cardiovascular disease [17]. Evidence suggests an increased likelihood of weight loss when multiple health behavior changes are implemented compared to one [10, 16, 18]. From a behavioral and motivational self-regulation standpoint, the synergistic effects of improving diet and physical activity have been investigated. A study from Mata et al. [19] showed that physical activity self-determination predicted eating self-regulation and fully mediated the relationship between physical activity and eating self-regulation during a lifestyle weight-management programme [19]. This suggests that psychological mechanisms involved in motivation may help explain the association between physical activity and eating behaviors. Nevertheless, there is a dearth of knowledge regarding the effects of multiple health behavior changes by exercise prescription patients to improve metabolic, physiological and psychological outcomes. This study aimed to analyse the impact of changing diet and increasing exercise on health improvements among exercise prescription patients.

## Methods

The ethics application for this study was considered and subsequently waived by the Health and Disability Ethics Committees in New Zealand due to the research being an evaluation of an existing programme. Responses were collected on an informed consent basis as part of the 17th annual Green Prescription patient survey. The survey was administered by Research New Zealand as contracted by the NZ Ministry of Health to measure the performance of Green Prescription.

This mixed-method online, telephone and paper-based survey was conducted from March-May 2016 using a stratified random sample. Green Prescription patients who had contact with one of the 17 Green Prescription contract holders in all District Health Boards over 6 months from July-December 2015 were eligible for sampling.

### Sample

Contract holders throughout New Zealand, who are responsible for delivering the national Green Prescription Programme, submitted their patient list to Research New Zealand, totaling 18,849 Green Prescription patients throughout the country. Historically, there have been lower survey response rates among minority groups enroled in Green Prescription, namely, Māori and Pacific. Assuming a low response rate, an oversampling of these groups was executed to help ensure a more ethnically-representative sample of patients. In the total sample, European New Zealander respondents comprised 59%, Māori 28% and Pacific 13%. The first step in the data collection process entailed separating larger contract holders (with > 700 patients) from smaller contract holders. A sample of *n* = 2440 Māori and Pacific patients was randomly selected from the combined lists of the larger contract holders, proportional to the total number of Māori and Pacific patients on these lists. All patients with known contact details on the lists of smaller contract holders (*n* = 4560) were also selected. Finally, a random sample (*n* = 3000) was selected from the remaining lists of the larger contract holders in proportion with the total number of non-Māori/Pacific patients.

On 7th March 2016, selected patients were sent a letter from Research New Zealand inviting them to participate, along with a paper copy of the survey, and a reply-paid envelope with three $250 gift vouchers used as incentive. The letter introduced the survey and its purpose and gave instructions for completing the survey on paper or online. On 30 March 2016, 4657 patients who had not yet responded were sent a reminder letter and 1052 were sent a reminder email. Commencing 30 April 2015, a reminder call was made to all non-responding Māori and Pacific patients (*n* = 1973), and non-Māori and Pacific patients (*n* = 960). Of these, 1478 were contacted during the reminder call period (each was called a maximum of five times). The main surveying period ended on 15 May 2016.

To account for the varying sampling criteria applied to large and small contract holders and the different participation rates, the results were weighted to be representative of the proportion of patients from each contract holder. The weighted results for the total sample have a maximum margin of error of plus or minus 1.8%, at the 95% confidence level (p. 15) [20].

### Participation rate

**Fig. 1:**
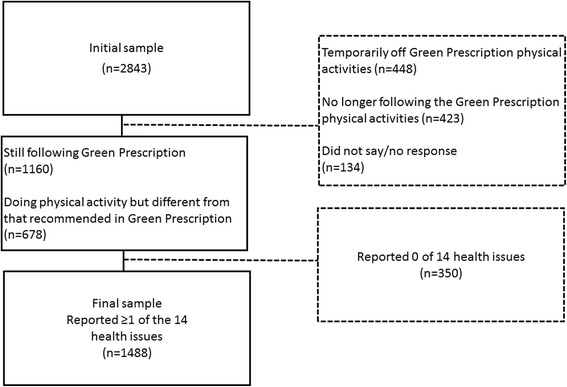
Flow diagram showing patient inclusion (box) and exclusion (dotted box) criteria for assessment

A representative sample of 10,000 patients were invited to complete the survey. A total of *n* = 2843 valid, completed responses were received during the survey period (*n* = 2045 paper, *n* = 496 online, and *n* = 302 telephone), representing a participation rate of 28% [20]. Data was screened according to the flow diagram in Fig. 1. Patients reporting they were temporarily off of (*n* = 448) or were no longer following Green Prescription physical activities (*n* = 423) and those who didn’t respond to this item (*n* = 134) were excluded from analysis. Those included in analysis were either still following Green Prescription physical activities (*n* = 1160) or they were engaging in a physical activity different from their Green Prescription recommendations (*n* = 678). Patients who reported receiving a Green Prescription for “heart problems” (*n* = 202), “injury/surgery recovery” (n = 202) and/or “other” (*n* = 258) were excluded from analysis. These reasons could have prevented or hindered patients’ ability to engage in physical activity. In total, “1488 surveys were analysed, comprising 17% of participants being first prescribed a Green Prescription less than 4 months ago, 28% 4-6 months ago, 22% 6-8 months ago and 33% more than 8 months ago.” Table 1 displays the sex, age and ethnicity of all patients used for analysis after the data screening.

**Table 1:** Frequencies and percentages of sex, age and ethnicity

	Number	Percent
Sex		
Male	428	29
Female	1049	71
Age		
Under 18	11	0.7
18-24	45	3.0
25-59	51	3.4
30-34	54	3.6
35-39	67	4.5
40-44	104	7.0
45-49	136	9.2
50-54	156	10.5
55-59	181	12.2
60-64	151	10.2
65-69	208	14.0
70-74	159	10.7
75-79	99	6.7
80 or older	60	4.0
Ethnicity		
New Zealand European	603	40.5
Māori	371	24.9
Samoan	43	2.9
Cook Island Māori	21	1.4
Tongan	32	2.2
Niuean	11	0.7
Chinese	14	0.9
Indian	32	2.2
Other Asian (e.g. Korean, Filipino)	9	0.6
Other Pacific (e.g. Tokelauan, Fijian)	16	1.1
British/European	72	4.8
Other	87	5.8

*N* = 1488

### Health problem subsamples

The survey instrument contained two key variables used for analysis; health problems and health improvements. As the independent variables, the health problems were identified in an item asking participants to choose one or more reasons they were written a Green Prescription. Patients who only selected “heart problems”, “injury/surgery recovery”, and/or “other”, were excluded from analysis since these problems were not clearly linked to the health improvement response options. The remaining 14 health problems were then categorised into one of seven subsamples; metabolic, physiological, psychological, sleep problems, asthma/breathing problems, fall prevention, or smoking. The top of Table 2 lists the frequencies of all health problems. The bottom of Table 2 indicates the number of health problems reported within the metabolic, physiological, and psychological subsamples containing five, three and two health problems, respectively. Health problems within these three subsamples were co-dependent or associated with others in the same subsample. For example, a participant reporting high blood pressure, risk of diabetes and high cholesterol would be considered in the metabolic subsample analysis to determine his/her likelihood of achieving any of the metabolic-related health improvements listed on Table 5. A patient reporting health problems of depression and high blood pressure was analysed in both the metabolic and the psychological subsamples to determine the likelihood of achieving the associated health improvements (i.e. lower blood pressure, feeling less depressed/anxious). Combining health problems into subsamples made for a more robust analysis.

**Table 2:** Frequencies and percentages of individual health problems and frequency of health problems within subsamples containing more than one health problem

Health problems	Number	Percent
Weight problems^a^	886	59.5
High blood pressure/risk of stroke^a^	424	28.5
Arthritis^b^	397	26.7
Back pain or problems^b^	382	25.7
Stress^c^	345	23.2
High cholesterol^a^	311	20.9
Depression/anxiety^c^	287	19.3
Diagnosed type 2 diabetes^a^	271	18.2
Sleep problems	253	17.0
Asthma/breathing problems	243	16.3
Pre-diabetes/risk of diabetes^a^	229	15.4
Fall prevention	104	7.0
Osteoporosis^b^	78	5.2
Smoking	67	4.5
Subsamples containing > 1 health problem		
1 Metabolic health problem	605	40.7
2 Metabolic health problems	332	22.3
3 Metabolic health problems	174	11.7
4 Metabolic health problems	75	5.0
5 Metabolic health problems	6	0.4
Total metabolic health problems	1192	80.1
1 Physiological health problem	427	28.7
2 Physiological health problems	170	11.4
3 Physiological health problems	30	2.0
Total physiological health problems	627	42.1
1 Psychological health problem	262	17.6
2 Psychological health problems	185	12.4
Total psychological health problems	447	30.0

^a^Metabolic health problem

^b^Physiological health problem

^c^Psychological health problem

### Measures

#### Health behaviour

The health behavior predictor variable was used to create four behaviour change groups for comparison; 1. increased physical activity, 2. changed diet (diet), 3. increased physical activity and changed diet (physical activity and diet), or 4. no changes to physical activity and diet (control group). Groupings were created by using responses from two items regarding behavior changes to physical activity and diet. The physical activity item was, “Compared with the time before you were first given a Green Prescription, are you now spending more time being active, about the same amount of time being active or less time being active?” Patients choosing the latter two options were combined into the group “no increase in physical activity.” The diet item was, “Have you made any changes to your food and/or drink intake since being given your Green Prescription?” and contained “yes” and “no” response options. Table 3 indicates the frequencies of health problems for all four behaviour change groups.

**Table 3:** Frequency of health problems by behavior change groups and control group (neither PA nor diet)

	Increased PA		Changed Diet		PA + Diet		Neither PA nor Diet		Total	
	n	%	n	%	n	%	n	%	N	%
Entire sample	258	17.3	268	18.0	825	55.4	137	9.2	1488	
Metabolic problems	160	13.4	219	18.4	723	60.7	90	7.6	1192	79.5
Physiological problems	121	19.3	127	20.3	302	48.2	77	12.3	627	42.1
Psychological problems	78	17.4	81	18.1	253	56.6	35	7.8	447	30.0
Sleep problems	45	17.8	44	17.4	145	57.3	19	7.5	253	17.0
Asthma/breathing	47	19.3	53	21.8	130	53.5	13	5.3	243	16.8
Fall prevention	21	20.2	19	18.3	46	44.2	18	17.3	104	7.2
Smoking	13	19.4	14	20.9	33	49.3	7	10.4	67	4.6

*N* = 1488. *PA* physical activity

#### Health improvements

There were 15 health improvements analysed as dependent variables. Patients who reported “yes” to noticing positive changes since first being issued a Green Prescription were then prompted to answer the follow-up item, “If yes, what positive changes have you noticed?” There were originally 19 response options, but the options “feel stronger/fitter”, “generally feel better”, “more energy”, and/or “other” were excluded from analysis as these options do not directly associate with any one particular health problem. Descriptive statistics of the 15 health improvements are listed in Table 4.

**Table 4:** Frequencies and percentages of health improvements noticed by Green Prescription patients

Health improvements	Number	Percent
Lost weight	618	45.9
Breathing easier	430	31.9
Less stressed	419	31.1
Sleeping better	347	25.8
Increased mobility	345	25.6
Less joint pain/discomfort	323	24.0
Less back pain	268	19.9
Feel less depressed/anxious	240	17.8
Lower blood pressure	240	17.8
Improved blood sugar levels	205	15.2
Lower cholesterol	177	13.1
Better balance/fewer falls	162	12.0
Fewer illnesses	128	9.5
Less medication	124	9.2
Smoking less	63	4.7

*N* = 1488

### Analysis

A predictive analysis was conducted through multinomial regression to interpret odds ratios (OR). A linear regression was calculated to test the assumption of multicollinearity. The minimum cut off for tolerance was set at 0.2 and the maximum cut off for the variance inflation factor (VIF) was 5. All independent variables met these assumptions, with tolerances ranging between .699 and .956 and VIF ranging between 1.430 and 1.046. All other assumptions for multinomial regression were met. Multinomial regressions were conducted using the health behavior groups as the predictors (physical activity, diet and physical activity and diet) each compared to the control group (neither physical activity nor diet). Then, odds ratios (OR) were calculated with 95% confidence intervals. All multinomial regressions controlled for sex and age groups (under 60, over 59).

## Results

Overall, weight problems were the most commonly reported health problems (*n* = 886, 60%), followed by high blood pressure/risk of stroke (*n* = 424, 29%), arthritis (*n* = 397, 27%), and back pain/problems (*n* = 382, 26%) (Table 2). The most commonly reported health improvements were weight loss (*n* = 618, 46%), breathing easier (*n* = 430, 32%), and less stress (*n* = 419, 31%) (Table 4). After controlling for sex and age, patients in the diet group were more likely to improve 3 of the 15 possible health problems listed, and the physical activity group improved 6 of 15, but the physical activity and diet group was more likely to improve 11 of 15 health problems compared to the control group (Table 5).

**Table 5:** Odds ratios and confidence intervals for health improvements experienced in subsamples by behavior change group compared to controls (no changes in diet or physical activity) controlled for sex and age

Subsamples analysed with associated health improvements	Increased PA versus no changes			Changed diet versus no changes			Increased PA + changed diet versus no changes		
	OR	95% CI		OR	95% CI		OR	95% CI	
		Lower	Upper		Lower	Upper		Lower	Upper
Metabolic subsample (*n* = 1192)									
Lost weight	5.22***	2.10	12.94	7.22***	2.96	17.59	17.47***	7.43	41.05
Lower blood pressure	1.71	0.69	4.26	2.44*	1.02	5.87	3.19**	1.41	7.18
Lower cholesterol	1.35	0.41	4.41	3.50*	1.18	10.38	3.50*	1.24	9.87
Improved blood sugar	1.99	0.71	5.61	2.02	0.73	5.60	3.79**	1.48	9.67
Less medications	1.45	0.29	7.40	3.28	0.73	14.76	4.17*	1.00	17.50
Fewer illnesses	2.20	0.46	10.48	2.71	0.60	12.31	4.91*	1.18	20.51
Physiological subsample (*n* = 627)									
Less back pain	2.25*	1.04	4.89	1.25	0.56	2.81	2.36*	1.16	4.80
Less joint pain/discomfort	2.63*	1.23	5.62	1.05	0.46	2.38	3.16***	1.57	6.36
Increased mobility	6.23***	2.30	16.84	2.76	0.98	7.77	6.61***	2.55	17.08
Psychological subsample (*n* = 447)									
Less stressed	2.35	0.92	6.00	1.84	0.72	4.71	3.24**	1.38	7.60
Feel less depressed/anxious	1.16	0.46	2.93	0.84	0.33	2.14	1.33	0.58	3.06
Sleep subsample (*n* = 253)									
Sleeping better	5.53*	1.31	23.28	3.70	0.89	15.42	3.50	0.94	13.03
Asthma/breathing subsample (*n* = 243)									
Breathing easier	9.34**	1.76	49.60	4.59	0.87	24.37	14.64***	2.94	72.78
Fall prevention subsample (*n* = 104)									
Better balance/fewer falls	1.52	0.36	6.48	1.59	0.33	7.66	1.27	0.34	4.71
Smoking subsample (*n* = 67)									
Smoking less	4.23	0.26	68.81	4.18	0.26	68.29	3.09	0.22	43.84

*p ≤ .05, **p ≤ .01, ***p < .001, *PA* physical activity

### Sex

After controlling for differences in age and health behavior, males who reported one or more metabolic health problems were 2.0 times more likely to lower blood pressure (95% CI = 1.4 to 2.7), 1.8 times more likely to lower cholesterol (95%CI = 1.3 to 2.6), 2.0 times more likely to improve blood sugar levels (95% CI = 1.4 to 2.8), and 1.6 times more likely to reduce medication (95%CI = 1.0 to 2.5) than their female counterparts. Males reporting sleep problems were 1.8 times more likely to improve their sleep (95%CI = 1.0 to 3.2) than their female counterparts. Males enrolled in Green Prescription for fall prevention were 2.5 times more likely to improve balance/reduce falls (95% CI = 1.0 to 6.4) than females. Odds ratios and confidence intervals for positive changes experienced in subsample by sex and age are on Table 6.

**Table 6:** Odds ratios and confidence intervals for positive changes experienced in subsample by sex and age (< 60 vs > 59)

Subsample analysed with associated health improvements	Sex			Age		
	OR	95% CI		OR	95% CI	
		Lower	Upper		Lower	Upper
Metabolic subsample (*n* = 1192)						
Lost weight	1.17	0.88	1.56	1.43**	1.10	1.86
Lower blood pressure	1.96***	1.43	2.69	0.71	0.52	0.97
Lower cholesterol	1.84***	1.30	2.62	1.18	0.83	1.68
Improved blood sugar	2.01***	1.44	2.79	1.02	0.74	1.42
Less medications	1.61*	1.04	2.49	1.23	0.79	1.89
Fewer illnesses	1.37	0.90	2.07	1.37	0.90	2.07
Physiological subsample (*n* = 627)						
Less back pain	1.47	0.98	2.19	1.92***	1.31	2.79
Less joint pain/discomfort	0.80	0.53	1.20	1.04	0.72	1.51
Increased mobility	1.17	0.78	1.75	1.21	0.83	1.77
Psychological subsample (*n* = 447)						
Less stressed	1.27	0.80	1.10	0.85	0.55	1.32
Feel less depressed/anxious	1.00	0.64	1.58	0.90	0.59	1.40
Sleep subsample (*n* = 253)						
Sleeping better	1.81*	1.02	3.24	1.60	0.91	2.81
Asthma/breathing subsample (*n* = 243)						
Breathing easier	1.56	0.78	3.13	0.97	0.53	1.80
Fall prevention subsample (*n* = 104)						
Better balance/fewer falls	2.54*	1.01	6.39	0.82	0.24	2.86
Smoking subsample (*n* = 67)						
Smoking less	0.70	0.24	2.14	0.38	0.10	1.55

*p ≤ .05, **p ≤ .01, ***p < .001

### Age

After controlling for differences in sex and health behaviors, patients under 60 years old who reported one or more metabolic health problems were 1.4 times more likely to lower blood pressure (95%CI = 1.1 to 1.9) than 60+ year olds. Patients under 60 years old who reported physiological health problems were 1.9 times more likely to reduce back pain (95%CI = 1.3 to 2.8) than patients 60+ years old.

### Metabolic subsample

There were 20 significant ORs for the 15 health improvements analysed after controlling for sex and age (Table 6). Comprising the largest subsample in the study, there were 1192 patients reporting one or more metabolic health problems. Only 20% of patients received a Green Prescription for reasons unrelated to metabolic health problems (*n* = 296). Physical activity group patients who reported one or more metabolic health problems were 5.2 times more likely to lose weight compared to controls (95%CI = 2.1 to 12.9).

Patients in the diet group of the metabolic subsample were 7.2 times more likely to lose weight (95% CI = 3.0 to 17.6), 2.4 times more likely to lower blood pressure (95% CI = 1.0 to 5.9), and 3.5 times more likely to lower cholesterol than controls (95% CI = 1.2 to 10.4).

Patients in the physical activity and diet group of the metabolic subsample were 17.5 times more likely to lose weight (95% CI = 7.4 to 41.1), 3.2 times more likely to lower blood pressure (95%CI = 1.4 to 7.2), 3.5 times more likely to lower cholesterol (95% CI = 1.2 to 9.9), 3.8 times more likely to improve blood sugar levels (95% CI = 1.5 to 9.8), 4.2 times more likely to reduce medication (95%CI = 1.0 to 17.5), and 4.9 times more likely to experience fewer illnesses than controls (95% CI = 1.18 to 20.5).

The increased physical activity group was no more likely to lower blood pressure and cholesterol than the control group (OR = 1.7, 1.4 respectively). Physical activity and diet patients were more likely to lower blood pressure and cholesterol (OR = 3.2, 3.5 respectively), and the differences were equal to or stronger than the odds resulting from diet alone (OR = 2.4, 3.5 respectively). All results are listed on Table 5.

### Physiological subsample

Patients reporting one or more of arthritis, osteoporosis, or back pain/problems were included in the physiological subsample. In this subsample, physical activity group patients were 2.3 times more likely to reduce back pain (95% CI = 1.0 to 4.9), 2.6 times more likely to reduce joint pain/discomfort (95% CI = 1.2 to 5.6), and 6.2 times more likely to increase mobility (95% CI = 2.3 to 16.8) than controls. Patients in the diet group showed no improvements compared to controls, but patients in the physical activity and diet group were 2.4 times more likely to reduce back pain (95%CI = 1.2 to 4.8), 3.2 times more likely to reduce joint pain/discomfort (95% CI = 1.6 to 6.4), and 6.6 times more likely to increase mobility than controls (95% CI = 2.6 to 17.1) (Table 5).

Changing diet did not change the odds of physiological improvements to back, joints and mobility as was observed in the physical activity group (OR = 2.4, 2.6, 6.2, respectively), and the likelihood was further increased in the physical activity and diet group (OR = 2.4, 3.2, 6.6 respectively).

### Other subsamples

Patients reporting stress or depression/anxiety were 3.2 times more likely to reduce stress than controls (95% CI = 1.4 to 7.6) if they were in the physical activity and diet group. Regarding sleep, patients in the physical activity group who reported sleep problems were 5.5 times more likely to improve sleep than controls (95% CI = 1.3 to 23.3). Among patients reporting asthma/breathing problems, the physical activity group was 9.3 times more likely to improve breathing than controls (95% CI = 1.8 to 49.6) while patients in the physical activity and diet group were 14.6 times more likely to improve breathing than controls (95% CI = 2.9 to 72.8). Increasing physical activity and/or changing diet did not change the odds of improving balance/having fewer falls or smoking less.

### Subsample comparisons

The results within the subsamples indicated that 80% of patients reported more than one metabolic health problem while 42% and 30% reported physiological and psychological health problems, respectively (Table 3). Of the five health problems in the metabolic subsample, changing diet alone significantly increased the odds of weight loss (OR 7.2), lower blood pressure (OR 2.4) and lower cholesterol (OR 3.5), whereas increasing physical activity alone led to weight loss (OR 5.2). The odds for weight loss were greatly increased in the physical activity and diet group (OR = 17.5).

## Discussion

The results of this study within a representative sample of the New Zealand exercise prescription patients are in accord with Clark’s study on overfat adults, [21] suggesting the necessity to include physical activity in combination with diet to elicit the highest likelihood of experiencing improvements in metabolic health problems. This study supports previous findings that reducing energy intake by changing diet while increasing energy expenditure through increased physical activity addresses both components of the energy balance equation and leads to considerably higher odds of losing weight [22]. Additionally, this study suggests that exercise prescription patients who increase physical activity without changing diet were successful in dealing with physiological problems, poor sleep, asthma, and weight loss compared to controls. Surprisingly, 18% of patients following a Green Prescription changed their diet without increasing physical activity and they revealed higher odds of weight loss, and lowering blood pressure and cholesterol. Nevertheless, the majority of exercise prescriptions patients analysed in this study (55%) reported increased physical activity levels while changing diet. Although nutrition is not formally delivered as part of the Green Prescription programme, findings from this study suggest most exercise prescription patients also change their dietary behaviours as well.

These results provide support that exercise prescription patients who change multiple energy balance behaviors can improve multi-factor health problems like metabolic syndrome and cardiovascular disease. Support for this exists in extant literature among adults [7–12] and youth [4]. Baker and Brownell [23] suggest that exercise influences both physiological processes such as energy metabolism and appetite as well as psychological aspects like self-efficacy, body image, or mood, improving the likelihood of long-term weight management. Moreover, they surmise that the latter mechanisms could lead to stronger motivation and confidence, which could improve eating self-regulation, dietary compliance and long-term exercise adherence [23]. Besides physiological effects of exercise, which may affect appetite regulation, motivational mechanisms may also explain the association between physical activity and eating behaviours. Future research should investigate whether physical activity can serve as a gateway behaviour for motivational changes in eating regulation among exercise prescription patients.

The findings could be interpreted in several ways. First, in order to amplify metabolic health improvements, exercise prescription patients might consider complementing their physical activity with changes to their diet. Second, an exercise prescription programme, designed to increase physical activity, also resulted in changes to diet. Third, the combined effects of diet and physical activity was associated with more health improvements than either behaviour change alone. These findings could be of major importance for health care systems, allowing savings of health care resources. The notion that physical activity could have synergistic effects in changing eating behaviors is a very powerful one, given the combined benefits found for several metabolic health indicators and the high incidence of those indicators in today’s society.

Although Green Prescription currently offers healthy eating information and tips to patients, some contract holders might offer a more specialised service with registered dieticians. Green Prescription, and possibly other exercise prescription programmes, might consider provisions for a nutritional component such as consultations with a registered dietitian. Understanding healthy eating and improving one’s diet would be well-suited for patients with metabolic health problems. Considering the evidence in this research and given that the majority of patients who follow the Green Prescription programme in this study (80%) were prescribed exercise for at least one metabolic health problem, Green Prescription funders should consider strengthening the dietary component of the programme, especially considering the strong association between diet and metabolism.

### Limitations

There were several limitations in this study. First, creating subsamples with four behavior groups each made for smaller group comparisons with less power. For example, no health behavior changes improved the odds of reporting better balance or less smoking compared to controls. This result could be due reduced numbers in each health behavior group or due to irrelevant associations from physical activity and/or diet for such health improvements. The subsamples were created to address the smaller group sizes. Second, self-report surveys are subject to misreporting and are not objective measures. Moreover, the item with the lost weight response option did not account for body composition. Third, the types of physical activity and dietary changes were not specified in the survey, i.e. endurance versus resistance training. Fourth, only 28% of the invited participants completed the survey. It is possible that these respondents were the most motivated and successful ones, potentially explaining the high rates of patients reporting changes to physical activity and diet. Fifth, a social desirability bias could have been present due to nature of the data collection and this could have been amplified by the fact that people received financial incentives to participate ($250 gift vouchers). Sixth, the vague nature of the questions used to assess physical activity and diet changes (“*are you now spending more time being active?”* and *“Have you made any changes to your food and/or drink intake?”)* impose the assumption that respondents have the same notion of what “more active” means and it is possible that respondents’ food and drink intake changed to became less healthy. Future Green Prescription surveys should improve these questions to quantify the responses in a clearer and more subjective manner. Finally, although the analyses controlled for the covariates sex and age, one cannot ignore the existence of other residual confounders.

## Conclusion

Exercise prescription patients who made behavior changes to diet and physical activity gained greater health improvements than those who changed only one behaviour. This suggests that undertaking two energy balance behavior changes within an exercise prescription programme can improve the likelihood of achieving health improvements. This study supports existing evidence [16] that changing one’s health status requires a manipulation of an elaborate network of interacting, complimenting, and confounding factors. Adding a nutrition component to physical activity prescription programmes may increase the potential for patients to experience improvements in metabolic, physical and psychological health, potentially enabling them to reverse the deleterious co-morbidities they are at risk of experiencing otherwise. Exercise prescription programmes would become more robust and can be further personalised in their delivery approach if dietary counselling is included as part of usual care. Further studies are needed to determine the impact and economic viability of incorporating a nutritional component to exercise prescription programmes [16].
